# Time Switching Based Wireless Powered Relay Transmission with Uplink NOMA

**DOI:** 10.3390/s21165467

**Published:** 2021-08-13

**Authors:** Zhihua Lin, Shihua Cao, Jianqing Li

**Affiliations:** 1 Faculty of Information Technology, Macau University of Science and Technology, Avenida Wai Long, Taipa 999078, Macau, China; lindiva@fjjxu.edu.cn; 2 College of Electronic and Information Science, Fujian Jiangxia University, Fuzhou 350108, China; 3 QianJiang College, Hangzhou Normal University, Hangzhou 310012, China; csh@hznu.edu.cn

**Keywords:** time switching, energy harvesting, relay cooperative communication, uplink NOMA, spectrum efficiency

## Abstract

Non-orthogonal multiple access (NOMA) utilizes power domain multiplexing to improve spectrum efficiency compared with orthogonal multiple access (OMA). In the Internet of Things (IoT) uplink NOMA networks, if the channel between the far-end node and the base station is in deep fading, allocating larger transmitting power for this node cannot achieve higher spectrum efficiency and overall system throughput. Relay cooperative communication reduces the transmitting power at the far-end node but leads to extra energy expenditure at the relay node. Fortunately, simultaneous wireless information and power transfer (SWIPT) is advocated in energy-constrained IoT networks to save energy consumption. However, early works all focus on energy harvesting (EH) from one source node or one dedicated power supply station. In this paper, we propose a time switching based wireless powered relay transmission model with uplink NOMA where our EH technique can harvest energy from two simultaneously transmitting nodes. More importantly, by optimizing relay position more energy is harvested from the near-end node at the relay and relay signal attenuation to the destination is reduced as well. Furthermore, the closed-form expressions of outage probability and overall system throughput are derived, and numerical results prove that NOMA in our EH scheme achieves better performance compared to the traditional EH scheme and OMA by optimizing the position of the relay node, time switching factor and so on.

## 1. Introduction

With tremendous deployment of machine-type nodes for the Internet of Things (IoT) applications in the coming years, wireless communication technology has faced severe scarcity of radio spectrum resources [1]. Conventional orthogonal multiple access (OMA) communication techniques, such as time division multiple access (TDMA), and orthogonal frequency division multiple access (OFDMA) require one orthogonal resource block to serve for one single node [2]. In a typical OFDMA scenario, a far-end node with poor channel condition has to be allocated enough subcarriers to meet its quality of service (QoS) requirement, while the near-end node with the perfect channel is forced to use the remaining narrow bandwidth, which may greatly reduce the spectrum utilization and overall system throughput. Unlike OMA, non-orthogonal multiple access (NOMA) utilizes power domain multiplexing to implement scarce spectrum resource sharing, where the transmitting signals of different nodes are assigned at different power levels but at the same time, frequency, and code [3]. For example, in a downlink NOMA scenario, a base station (BS) serving for multiple nodes simultaneously will assign a power ratio for each node in a superimposed transmitting signal. The key principle of NOMA is that more power is allocated for the far-end node, and less power for the near-end node in a given total power constraint since long distance transmission will cause the larger path loss. After receiving the superimposed signal, the near-end node first decodes the signal of the far-end node, and then decodes its own signal by successive interference cancellation (SIC), while the far-end node only decodes its high-power signal by treating other signals as interference. In an uplink NOMA scenario, all the messages from different nodes are transmitted simultaneously to the BS in the same frequency band. The strong signal, which may belong to the near-end node or the good channel condition, will be first decoded at the BS. The weak signal from the far-end node is decoded at last. Therefore, NOMA can achieve higher spectrum efficiency, connectivity and fairness, which is significant for 5G wireless networks to support IoT functionalities.

In terms of NOMA system performance, superior outage probability (OP) and sum rate are demonstrated by optimizing the power allocation scheme compared with OMA [3,4]. The work in [5] has further investigated NOMA performance in cognitive radio networks to guarantee QoS of the nodes with poor channel conditions. However, if the channel between the far-end node and the BS is in deep fading, larger energy allocation for this node at downlink NOMA is unable to achieve high spectrum efficiency and overall system throughput, but increase energy consumption. Moreover, it is unfeasible for a battery-operated IoT node to consume enormous transmitting power in uplink NOMA. Fortunately, relay cooperative communication has been widely employed to cope with wireless channel impairments, e.g., fading and interference [6,7]. For example, in a relay cooperative network, if no direct link exists between the source and the destination, with the aid of relay nodes, the transmission distance per hop is shortened. Due to lower path loss and lower interference, transmitting power is also reduced. Furthermore, cooperative communication may enhance the signal-to-noise ratio (SNR) due to two copies received at the destination if the destination can also receive the message from the source directly.

Relay cooperative communication techniques have been introduced to NOMA in [8,9,10,11]. Some suboptimal power allocation strategies are proposed and system performance, e.g., OP and achievable average rate, is analyzed in the cooperative relay NOMA system [8,9]. The work [10] adopts user pairing selection opportunistically to operate in direct NOMA mode or cooperative NOMA mode to guarantee transmission reliability and fairness. Additionally, an adaptive switching approach between OMA mode and cooperative NOMA mode is realized to improve the far-end node’s performance in [11].

Although cooperative relaying techniques increase overall system performance, forwarding also leads to extra energy expenditure at the relay node, which may prevent energy-constrained IoT nodes from participating in relaying operation [12]. Fortunately, energy harvesting (EH) techniques have emerged to reduce energy consumption in energy-constrained wireless IoT or sensor networks [13]. In particular, a new promising solution to harvest energy from ambient radio frequency (RF) signals is encouraged in wireless networks since RF signals can carry both power and information at the same time, thus those energy-constrained nodes can obtain energy and decode the information simultaneously. In a simultaneous wireless information and power transfer (SWIPT) system, an IoT relay node can scavenge energy and receive information from the wireless signals of the source node, and then use the harvested energy to realize relay forwarding. There are two main paradigms to implement wireless powered relay (WPR): time switching (TS) relay and power splitting (PS) relay [14]. In TS, one source node transmits RF signals to the relay node in β (TS factor) period for EH and the remaining 1-β period for information reception and forwarding. In PS, a portion δ (PS factor) of signal power received from one source node is used for EH, while the rest 1-δ is used for information decoding. The literature [15,16] investigates PS-based NOMA communication, while the achievable rate and OP in IoT relay NOMA system are analyzed under TS architecture [12,17].

Theoretically, OMA may be more suitable than NOMA in downlink communication, as it can gain higher system throughput [18]. Moreover, it is difficult for a tiny battery-operated IoT node to realize multi-user detection (MUD) in downlink NOMA decoding [19]. To the best of our knowledge, most of the literature only focuses on power allocation in downlink communication from BS to IoT nodes. However, in practice, it is more common in IoT applications, e.g., smart home, smart farming, and intelligent transportation that the IoT nodes, e.g., sensors or cameras, transmit their gathered data to BS or sink node via an uplink channel. Therefore, uplink NOMA is more worthy of investigation in wireless IoT networks. In the related works about uplink NOMA, the authors in [20] only plot energy efficiency in a direct downlink and uplink NOMA system, while the authors in [21] exploit relay uplink NOMA without taking wireless EH into account. Considering wireless EH in relay cooperative networks is a hot research topic, our paper focuses on a wireless powered relay system with uplink NOMA in IoT networks to improve spectrum efficiency and reduce energy consumption, which has never been investigated as far as we know. Different from the traditional EH scheme where the relay harvests energy only from one source or one dedicated power supply station, in our uplink NOMA model, the relay node can harvest RF power from two transmitting nodes due to power multiplexing transmission based on NOMA. Meanwhile, proper relay selection scheme can also reduce relay signal attenuation to improve the performance of the far-end node. In addition, in terms of selecting PS or TS scheme, PS is more complicated and expensive than TS for it uses a power splitter hardware component [22], which is not suitable for low-cost IoT nodes. Additionally, compared with OMA, the PS factor δ is more difficult to optimize due to complex superimposed signals in uplink NOMA. Therefore, TS scheme is employed in our wireless powered cooperative relay model with uplink NOMA.

In this paper, we aim to investigate system performance under different parameters, e.g., TS factor, power allocation factor, the position of relay node, and total transmitting power. Moreover, we also prove our proposed EH scheme achieves superior performance compared with the traditional EH scheme and OMA by numerical results.

The contributions of this paper are as follows:

- We investigate a relay based uplink NOMA model where the relay can harvest energy from two simultaneously transmitting nodes, i.e., far-end node and near-end node, thanks to the nature of uplink NOMA. As far as we know, this model has never been exploited. Based on our proposed model, the relay close to near-end node not only harvests more energy from near-end node via power allocation factor but also forwards the signal via a shorter path between relay and destination, which reduces path attenuation greatly and achieves higher energy and spectrum efficiency. It is unfeasible in the traditional EH scheme because the relay closer to the source has to be chosen in order to harvest higher forwarding power regardless of long distance relay signal fading. Furthermore, it is impossible to deploy one dedicated power supply station to improve energy efficiency because the station is fixed, high-cost, and low energy-efficient for the far-end node in massive IoT networks;
- We analyze EH from the superimposed signal and NOMA decoding at different cases in detail. Meanwhile, the closed-form expressions of outage probability and overall system throughput at different communication phases are derived for further numerical simulations;
- Our numerical analysis provides practical insights into the impacts under different system parameters. Superior system performance is achieved by optimizing the position of relay, TS factor, power allocation factor, etc. Additionally, simulation results also prove that NOMA with our proposed EH scheme outperforms that with the traditional EH scheme and OMA.

The rest of this paper is organized as follows. Section 2 introduces the system model of uplink NOMA. Closed-form expressions about outage probability and system throughput are derived in Section 3. Section 4 conducts numerical simulations and results analysis. Finally, Section 5 concludes the paper.

## 2. System Model

### 2.1. Model Introduction

**Figure 1:**
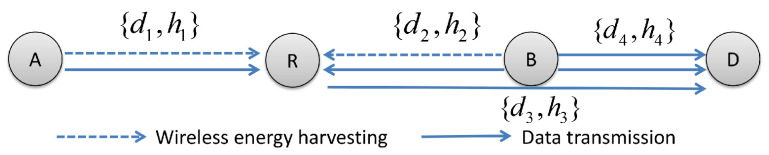
Wireless powered relay system with uplink NOMA.

In our considered system model (see Figure 1), node A and node B are IoT nodes or sensors, which both intend to transmit their messages to the BS D with uplink NOMA to improve total spectrum efficiency. Due to long distance and deep fading between A and D, we assume that there is no direct link between them, and the relay R in the midst of A and B assists A to deliver the message to D. Considering that R is an energy-constrained node, and relay forwarding will consume additional energy expenditure, a TS relay protocol [14] is employed at R to scavenge energy from A and B.

We define $d_{i}$ and $h_{i}$, i∈{1,2,3,4}, as distance and channel coefficient between two nodes, respectively, shown in Figure 1. We assume $h_{i}∼$*CN*(0, 1) follows small scale Rayleigh fading. In addition, we also assume each node in our communication model is equipped with one single antenna.

There are three phases to perform TS relay uplink NOMA transmission. For simplicity, the whole transmission duration is set as a unity. Moreover, a total transmitting power for this uplink NOMA transmission is given as *p*, hence node A and node B at different communication phases are allocated different transmitting power under a total power constraint. It is noted that the total power constraint is a critical criterion. Firstly, OMA allocates all power for one user at a certain resource block, while NOMA requires a user pair to implement power domain multiplexing. As a whole of nodes A and B, it is rational to limit the total power sum. Secondly, judging system performance between NOMA and OMA requires a same total power constraint. Lastly, in a cluster where multiple users share the same frequency band, total power constraint is necessary to reduce inter-cluster interference.

Phase 1: During the beginning of β fraction transmission period, A sends a non-information signal $x_{a}$ to R for wireless EH. Meanwhile, B transmits its own information-bearing signal $x_{b}$ to D. Both of them use their respective power allocation factors ($w_{1}$ for A, $w_{2}$ for B and $w_{1}+w_{2}=1$). Thus, relay node R will receive a combined signal for EH. Additionally, due to no direct link between A and D, D can only receive and decode the signal from B.

Phase 2: A sends an information-bearing signal $x_{a}$ to R with $w_{3}$ ratio of the given power *p* during the (1−β)/2 fraction of a unity transmission period, and B concurrently transmits its own signal $x_{b}$ to D with $w_{4}$ ratio of power *p*. Similarly, we have $w_{3}+w_{4}=1$. After receiving the combined signal, the relay R uses the NOMA scheme to decode the signal of A, while D decodes the signal of B regularly.

Phase 3: At the rest (1−β)/2 period, R forwards the signal decoded from A with the energy harvested at phase 1, and B continues transmitting its own message to D. At this phase, B uses the total power *p*. After receiving the superimposed signal from B and R, D first decodes the stronger signal from B due to the shorter distance between B and D treating the signal from R as interference, and then decodes the signal from R after removing the signal of B in the superimposed signal.

### 2.2. NOMA Communication Process Analysis

#### 2.2.1. Phase 1

At phase 1, the signal received at R is given by

(1) $$y_{r}^{1}=\frac{h_{1}\sqrt{w_{1}p}x_{a}}{\sqrt{{d_{1}}^{\alpha }+1}}+\frac{h_{2}\sqrt{w_{2}p}x_{b}}{\sqrt{{d_{2}}^{\alpha }+1}}+n_{r}$$

where α represents the path loss exponent, and $n_{r}$ denotes additive white Gaussian noise (AWGN) at R with variance $\sigma^{2}$. Furthermore, for simplifying our analysis, we assume the variances of noise in all nodes are equal. It is worth noting that we use the bounded path loss model in (1) to ensure that the path loss is always larger than one for any given distance [23], which is suitable for short transmission communication or dense urban scenarios in our considered IoT network.

By defining $c_{i}={d_{i}}^{\alpha }+1,i\in \left\{1,2,3,4\right\}$, (1) is rewritten by

(2) $$y_{r}^{1}=\frac{h_{1}\sqrt{w_{1}p}x_{a}}{\sqrt{c_{1}}}+\frac{h_{2}\sqrt{w_{2}p}x_{b}}{\sqrt{c_{2}}}+n_{r}.$$

The energy harvested from two signals at R is written by

(3) $$E_{r}^{}=\eta \beta \left(\frac{|h_{1}{|}^{2}w_{1}p}{c_{1}}+\frac{|h_{2}{|}^{2}w_{2}p}{c_{2}}\right)$$

where η is energy conversion coefficient, β is TS factor in TS relay protocol.

By defining $X=\frac{|h_{1}{|}^{2}}{c_{1}}$, $Y=\frac{|h_{2}{|}^{2}}{c_{2}}$, and $V=w_{1}X+w_{2}Y$, we have

(4) $$E_{r}^{}=\eta \beta pV.$$

The destination D can only receive the signal $x_{b}$ at this phase, which is written by

(5) $$\begin{array}{c} y_{d}^{1}=\frac{h_{4}\sqrt{w_{2}p}x_{b}}{\sqrt{{d_{4}}^{\alpha }+1}}+n_{d}\\ \;\;=\frac{h_{4}\sqrt{w_{2}p}x_{b}}{\sqrt{c_{4}}}+n_{d} \end{array}$$

where $n_{d}$ denotes AWGN at D with variance $\sigma^{2}$.

Therefore, the SNR of $x_{b}$ at D is

(6) $$\begin{array}{cc} \gamma_{b-d}^{1} & =\frac{w_{2}pW}{\sigma^{2}}\\ & =w_{2}\rho W \end{array}$$

where $\rho =\frac{p}{\sigma^{2}}$ and $W=\frac{|h_{4}{|}^{2}}{c_{4}}$.

#### 2.2.2. Phase 2

At phase 2, R receives two signals from A and B, thus the superimposed signal is given by

(7) $$\begin{array}{cc} y_{r}^{2} & =\frac{h_{1}}{\sqrt{{d_{1}}^{\alpha }+1}}\sqrt{w_{3}p}x_{a}+\frac{h_{2}}{\sqrt{{d_{2}}^{\alpha }+1}}\sqrt{w_{4}p}x_{b}+n_{r}\\ & =\frac{h_{1}}{\sqrt{c_{1}}}\sqrt{w_{3}p}x_{a}+\frac{h_{2}}{\sqrt{c_{2}}}\sqrt{w_{4}p}x_{b}+n_{r}. \end{array}$$

In order to investigate NOMA decoding for the combined signal at R, there are two cases that need to be considered, case 1: X>Y, i.e., $\frac{|h_{1}{|}^{2}}{c_{1}}>\frac{|h_{2}{|}^{2}}{c_{2}}$ and case 2: X<Y, i.e., $\frac{|h_{1}{|}^{2}}{c_{1}}<\frac{|h_{2}{|}^{2}}{c_{2}}$.

It is worth noting that since *X* and *Y* are two independent variables, it is difficult to distinguish the two cases. We expect X>Y if $c_{1}<c_{2}$ or $d_{1}<d_{2}$, and X<Y if $c_{1}>c_{2}$ or $d_{1}>d_{2}$. Though $d_{1}<d_{2}$ cannot guarantee X>Y, and vice versa, it is a simple and efficient scheme to adopt the assumption under statistical channel state information [24].

Case 1: X>Y ($d_{1}<d_{2}$), R decodes $x_{a}$ due to its stronger signal by treating $x_{b}$ as interference, so the SNR of $x_{a}$ is given by

(8) $$\begin{array}{cc} \gamma_{a-r}^{2} & =\frac{w_{3}pX}{w_{4}pY+\sigma^{2}}\\ & =\frac{w_{3}\rho X}{w_{4}\rho Y+1}. \end{array}$$

Case 2: X<Y ($d_{1}>d_{2}$), R first decodes $x_{b}$ due to its stronger signal and treats $x_{a}$ as interference. The signal $x_{a}$ is obtained only after $x_{b}$ is successfully decoded and removed from the combined signal.

Hence, the signal to interference plus noise ratio (SINR) of $x_{b}$ is written by

(9) $$\gamma_{b-r}^{2}=\frac{w_{4}\rho Y}{w_{3}\rho X+1}.$$

The SNR of $x_{a}$ after removing the strong signal $x_{b}$ can be expressed by

(10) $$\gamma_{a-r}^{2}=w_{3}\rho X.$$

Additionally, the signal of B received at D at this phase is given by

(11) $$y_{d}^{2}=\frac{h_{4}}{\sqrt{c_{4}}}\sqrt{w_{4}p}x_{b}+n_{d}.$$

Hence, the SNR of $x_{b}$ at D is

(12) $$\begin{array}{cc} \gamma_{b-d}^{2} & =\frac{w_{4}pW}{\sigma^{2}}\\ & =w_{4}\rho W. \end{array}$$

#### 2.2.3. Phase 3

At phase 3, R uses the energy harvested at phase 1 to forward the signal $x_{a}$ to D. The transmitting power is given by

(13) $$\begin{array}{cc} P_{r}= & \frac{E_{r}}{(1-\beta )/2}\\ & =\frac{2\eta \beta pV}{1-\beta }. \end{array}$$

At the same time, B continues to transmit its signal to D. The combined signal at D is

(14) $$\begin{array}{cc} y_{d}^{3} & =\frac{h_{3}}{\sqrt{{d_{3}}^{\alpha }+1}}\sqrt{P_{r}}x_{a}+\frac{h_{4}}{\sqrt{{d_{4}}^{\alpha }+1}}\sqrt{p}x_{b}+n_{d}\\ & =\frac{h_{3}}{\sqrt{c_{3}}}\sqrt{2\eta \beta pV/(1-\beta )}x_{a}+\frac{h_{4}}{\sqrt{c_{4}}}\sqrt{p}x_{b}+n_{d}. \end{array}$$

We assume the signal from B is stronger than from R due to the shorter distance between B and D, the SNR of $x_{b}$ at D is given by

(15) $$\begin{array}{cc} \gamma_{b-d}^{3} & =\frac{pW}{P_{r}Z+\sigma^{2}}\\ & =\frac{\rho (1-\beta )W}{2\eta \beta \rho VZ+(1-\beta )} \end{array}$$

where $Z=\frac{|h_{3}{|}^{2}}{{d_{3}}^{\alpha }+1}=\frac{|h_{3}{|}^{2}}{c_{3}}$.

The SNR of the signal from R is

(16) $$\begin{array}{cc} \gamma_{r-d}^{3} & =\frac{P_{r}Z}{\sigma^{2}}\\ & =\frac{2\eta \beta \rho VZ}{1-\beta }. \end{array}$$

Based on the expressions of SNR above, we can further analyze the system performance of our proposed model.

## 3. Performance Analysis

### 3.1. Outage Probability of Link B->D

We define $r_{a}$ and $r_{b}$ as the required target rate (RTR) of the link A->D via R and B->D, respectively, thus, if the effective transmission rate of A->D or B->D is less than the given $r_{a}$ or $r_{b}$, link outage occurs. For instance, at phase 1, to avoid an outage from B to D, the following requirement should be satisfied,

(17) $$\log_{2}\left(1+\gamma_{b-d}^{1}\right)\geq r_{b}.$$

Namely,

(18) $$\gamma_{b-d}^{1}\geq e_{b}$$

where $e_{b}^{}=2^{r_{b}}-1$.

Hence, we have the outage probability (OP) of B->D at phase 1

(19) $$P_{b-d}^{1}=\mathrm{Pr}\left\{\gamma_{b-d}^{1}<e_{b}^{}\right\}.$$

Substituting (6) into (19), we have

(20) $$P_{b-d}^{1}=\mathrm{Pr}\left\{W<\frac{e_{b}^{}}{w_{2}\rho }\right\}.$$

Due to $h_{4}∼$*CN*(0, 1) and $W=\frac{|h_{4}{|}^{2}}{c_{4}}$, the path gain *W* is exponentially distributed random variable, i.e., W∼*E*($\frac{1}{c_{4}}$). Similarly, we have X∼*E*($\frac{1}{c_{1}}$), Y∼*E*($\frac{1}{c_{2}}$) and Z∼*E*($\frac{1}{c_{3}}$). Accordingly, the probability density functions (PDFs) of *X*, *Y*, *Z*, and *W* are given by $f_{X}\left(x\right)=c_{1}e^{-c_{1}x}$, $f_{Y}\left(y\right)=c_{2}e^{-c_{2}y}$, $f_{Z}\left(z\right)=c_{3}e^{-c_{3}z}$ and $f_{W}\left(w\right)=c_{4}e^{-c_{4}w}$ respectively.

Based on the PDF of *W*, (20) can be further expressed by

(21) $$\begin{array}{c} P_{b-d}^{1}=\int_{0}^{\frac{e_{b}^{}}{w_{2}\rho }}f_{W}\left(w\right)dw\\ \;\;=1-e^{-\frac{e_{b}^{}c_{4}}{w_{2}\rho }}. \end{array}$$

At phase 2, the SNR of B should satisfy the requirement $\gamma_{b-d}^{2}\geq e_{b}$, therefore, the OP of B->D is written by

(22) $$P_{b-d}^{2}=\mathrm{Pr}\left\{\gamma_{b-d}^{2}<e_{b}^{}\right\}.$$

By substituting (12) into (22), and some manipulations as (20) and (21), we have

(23) $$\begin{array}{c} P_{b-d}^{2}=1-e^{-\frac{e_{b}^{}c_{4}}{w_{4}\rho }}. \end{array}$$

At phase 3, the OP of B->D is given by

(24) $$P_{b-d}^{3}=\mathrm{Pr}\left\{\gamma_{b-d}^{3}<e_{b}^{}\right\}.$$

By substituting (15) into (24), and utilizing the PDFs of *W* and *Z*,

(25) $$\begin{array}{cc} P_{b-d}^{3} & =\mathrm{Pr}\left\{\frac{\rho (1-\beta )W}{2\eta \beta \rho VZ+(1-\beta )}<e_{b}^{}\right\}\\ & =1-c_{3}e^{-\frac{e_{b}^{}c_{4}}{\rho }}\int_{0}^{\infty }\frac{1-\beta }{2e_{b}^{}\eta \beta c_{4}v+\left(1-\beta \right)c_{3}}f_{V}\left(v\right)dv \end{array}$$

where $f_{V}\left(v\right)$ is the PDF of $V=w_{1}X+w_{2}Y$.

We have the following Theorem 1.

**Theorem** **1.** *The PDF of V is expressed as follows.*

$$f_{V}\left(v\right)=\left\{\begin{array}{cc} \frac{c_{1}c_{2}}{c_{2}w_{1}-c_{1}w_{2}}\left(e^{\frac{-c_{1}v}{w_{1}}}-e^{\frac{-c_{2}v}{w_{2}}}\right) & if\;c_{2}w_{1}-c_{1}w_{2}\neq 0\\ \frac{c_{1}c_{2}v}{w_{1}w_{2}}e^{-\frac{c_{2}v}{w_{2}}} & if\;c_{2}w_{1}-c_{1}w_{2}=0 \end{array}\right.$$

**Proof.**  See Appendix A. □

According to Theorem 1, at $c_{2}w_{1}-c_{1}w_{2}\neq 0$, (25) can be further calculated as

(26) $$\begin{array}{c} P_{b-d}^{3}=1-\frac{c_{1}c_{2}}{\left(c_{2}w_{1}-c_{1}w_{2}\right)}e^{-\frac{e_{b}^{}c_{4}}{\rho }}\times \underset{\left[I\right]}{\underbrace{\int_{0}^{\infty }\frac{1}{2e_{b}^{}\eta \beta c_{4}v/\left(1-\beta \right)c_{3}+1}\left(e^{\frac{-c_{1}}{w_{1}}v}-e^{\frac{-c_{2}}{w_{2}}v}\right)\;\;dv}}. \end{array}$$

Define $t=\frac{2e_{b}^{}\eta \beta c_{4}v}{\left(1-\beta \right)c_{3}}+1$,

(27) $$\begin{array}{cc} \left[I\right] & =\frac{\left(1-\beta \right)c_{3}}{2e_{b}^{}\eta \beta c_{4}}\times \;e^{\frac{\left(1-\beta \right)c_{1}c_{3}}{2e_{b}^{}\eta \beta w_{1}c_{4}}}\int_{1}^{\infty }\frac{1}{t}e^{-\frac{\left(1-\beta \right)c_{1}c_{3}}{2e_{b}^{}\eta \beta w_{1}c_{4}}t}\;\;dt-\frac{\left(1-\beta \right)c_{3}}{2e_{b}^{}\eta \beta c_{4}}\times e^{\frac{\left(1-\beta \right)c_{2}c_{3}}{2e_{b}^{}\eta \beta w_{2}c_{4}}}\int_{1}^{\infty }\frac{1}{t}e^{-\frac{\left(1-\beta \right)c_{2}c_{3}}{2e_{b}^{}\eta \beta w_{2}c_{4}}t}\;\;dt\;\;\;\;\;\;\;\;\;\;\;\;\;\;\\ & =\frac{\left(1-\beta \right)c_{3}}{2e_{b}^{}\eta \beta c_{4}}\times e^{\frac{\left(1-\beta \right)c_{1}c_{3}}{2e_{b}^{}\eta \beta w_{1}c_{4}}}E_{1}\left(\frac{\left(1-\beta \right)c_{1}c_{3}}{2e_{b}^{}\eta \beta w_{1}c_{4}}\right)-\frac{\left(1-\beta \right)c_{3}}{2e_{b}^{}\eta \beta c_{4}}\times e^{\frac{\left(1-\beta \right)c_{2}c_{3}}{2e_{b}^{}\eta \beta w_{2}c_{4}}}E_{1}\left(\frac{\left(1-\beta \right)c_{2}c_{3}}{2e_{b}^{}\eta \beta w_{2}c_{4}}\right) \end{array}$$

where $E_{1}\left(x\right)=\int_{1}^{\infty }\frac{e^{-xt}}{t}dt$ is the exponential integral.

Substituting (27) into (26), at $c_{2}w_{1}-c_{1}w_{2}\neq 0$

(28) $$\begin{array}{c} P_{b-d}^{3}=1-\frac{\left(1-\beta \right)c_{1}c_{2}c_{3}e^{-\frac{e_{b}^{}c_{4}}{\rho }}}{2e_{b}^{}\eta \beta c_{4}\left(c_{2}w_{1}-c_{1}w_{2}\right)}\times \left[e^{\frac{\left(1-\beta \right)c_{1}c_{3}}{2e_{b}^{}\eta \beta w_{1}c_{4}}}E_{1}\left(\frac{\left(1-\beta \right)c_{1}c_{3}}{2e_{b}^{}\eta \beta w_{1}c_{4}}\right)-e^{\frac{\left(1-\beta \right)c_{2}c_{3}}{2e_{b}^{}\eta \beta w_{2}c_{4}}}E_{1}\left(\frac{\left(1-\beta \right)c_{2}c_{3}}{2e_{b}^{}\eta \beta w_{2}c_{4}}\right)\right]. \end{array}$$

Similarly, at $c_{2}w_{1}-c_{1}w_{2}=0$,

(29) $$\begin{array}{c} P_{b-d}^{3}=1-\frac{\left(1-\beta \right)c_{1}c_{2}c_{3}}{2e_{b}^{}\eta \beta c_{4}w_{1}w_{2}}e^{-\frac{e_{b}^{}c_{4}}{\rho }}\times \int_{0}^{\infty }\frac{v}{v+\left(1-\beta \right)c_{3}/2e_{b}^{}\eta \beta c_{4}}e^{-\frac{c_{2}v}{w_{2}}}dv. \end{array}$$

According to $\int_{0}^{\infty }\frac{x}{x+\beta }e^{-\mu x}=\beta e^{\mu \beta }E_{i}\left(-\mu \beta \right)\;+\;\frac{1}{\mu }$,

(30) $$\begin{array}{c} P_{b-d}^{3}=1-\frac{\left(1-\beta \right)c_{1}c_{2}c_{3}e^{-\frac{e_{b}^{}c_{4}}{\rho }}}{2e_{b}^{}\eta \beta c_{4}w_{1}w_{2}}\times \left[\frac{\left(1-\beta \right)c_{3}e^{\frac{\left(1-\beta \right)c_{2}c_{3}}{2e_{b}^{}\eta \beta w_{2}c_{4}}}}{2e_{b}^{}\eta \beta c_{4}}E_{i}\left(-\frac{\left(1-\beta \right)c_{2}c_{3}}{2e_{b}^{}\eta \beta w_{2}c_{4}}\right)+\frac{w_{2}}{c_{2}}\right] \end{array}$$

where $E_{i}\left(\cdot \right)$ denotes the exponential integral function [25].

To summarize, the OP of B->D at phase 3 $P_{b-d}^{3}$ is given by (31).

(31) $$P_{b-d}^{3}=\left\{\begin{array}{c} 1-\frac{\left(1-\beta \right)c_{1}c_{2}c_{3}e^{-\frac{e_{b}^{}c_{4}}{\rho }}}{2e_{b}^{}\eta \beta c_{4}\left(c_{2}w_{1}-c_{1}w_{2}\right)}\times \;\;\left[e^{\frac{\left(1-\beta \right)c_{1}c_{3}}{2e_{b}^{}\eta \beta w_{1}c_{4}}}E_{1}\left(\frac{\left(1-\beta \right)c_{1}c_{3}}{2e_{b}^{}\eta \beta w_{1}c_{4}}\right)-e^{\frac{\left(1-\beta \right)c_{2}c_{3}}{2e_{b}^{}\eta \beta w_{2}c_{4}}}E_{1}\left(\frac{\left(1-\beta \right)c_{2}c_{3}}{2e_{b}^{}\eta \beta w_{2}c_{4}}\right)\right]\;\;\;\;\;\;\;\;\;\;\;\;\;\;\;\;\;if\;\;c_{2}w_{1}-c_{1}w_{2}\neq 0\\ 1-\frac{\left(1-\beta \right)c_{1}c_{2}c_{3}}{2e_{b}^{}\eta \beta c_{4}w_{1}w_{2}}e^{-\frac{e_{b}^{}c_{4}}{\rho }}\left[\frac{\left(1-\beta \right)c_{3}}{2e_{b}^{}\eta \beta c_{4}}e^{\frac{\left(1-\beta \right)c_{2}c_{3}}{2e_{b}^{}\eta \beta w_{2}c_{4}}}E_{i}\left(-\frac{\left(1-\beta \right)c_{2}c_{3}}{2e_{b}^{}\eta \beta w_{2}c_{4}}\right)+\frac{w_{2}}{c_{2}}\right]\;\;\;\;\;\;\;\;\;\;\;\;\;\;\;\;\;\;\;\;\;\;\;\;\;\;\;\;\;\;\;\;\;\;\;\;\;\;\;\;\;\;\;\;\;\;\;\;\;\;\;\;\;\;\;\;\;\;\;\;\;\;\;\;\;\;\;\;\;\;\;\;if\;\;c_{2}w_{1}-c_{1}w_{2}=0 \end{array}\right.\;\;$$

### 3.2. Outage Probability of Link A->D via R

In this subsection, we plot the OP of A->D via R. Due to relay communication, the OP of A->R and R->D should be derived first before we obtain the OP of the whole link A->D.

#### 3.2.1. Outage Probability of Link A->R

In order to obtain the OP of A->R, we consider two cases according to the position of R, i.e., $d_{1}<d_{2}$ and $d_{1}>d_{2}$.

Case 1: when $X>Y\;(d_{1}<d_{2})$, we have the OP of A->R

(32) $$P_{a-r}^{1}=\mathrm{Pr}\left\{\gamma_{a-r}^{2}<e_{a}^{}\right\}$$

where $e_{a}^{}=2^{2r_{a}^{}/\left(1-\beta \right)}-1$.

By substituting (8) into (32),

(33) $$P_{a-r}^{1}=\mathrm{Pr}\left\{X<\frac{e_{a}^{}w_{4}}{w_{3}}Y+\frac{e_{a}^{}}{w_{3}\rho }\right\}.$$

Considering X>Y and $X<\frac{e_{a}^{}w_{4}}{w_{3}}Y+\frac{e_{a}^{}}{w_{3}\rho }$, if $w_{3}-e_{a}^{}w_{4}>0$,

(34) $$P_{a-r}^{1}=\int_{0}^{\frac{e_{a}^{}}{\rho (w_{3}-e_{a}^{1}w_{4})}}\int_{y}^{\frac{e_{a}^{}w_{4}}{w_{3}}y+\frac{e_{a}^{}}{w_{3}\rho }}f_{X}\left(x\right)dxf_{Y}\left(y\right)dy.$$

Apply the PDFs of *X* and *Y*, and some integral operations,

(35) $$\begin{array}{cc} P_{a-r}^{1} & =\int_{0}^{\frac{e_{a}^{}}{\rho (w_{3}-e_{a}^{}w_{4})}}\left(e^{-c_{1}y}-e^{-\frac{c_{1}e_{a}^{}w_{4}}{w_{3}}y-\frac{c_{1}e_{a}^{}}{w_{3}\rho }}\right)c_{2}e^{-c_{2}y}dy\\ & =\frac{c_{2}}{c_{1}+c_{2}}-\frac{w_{3}c_{2}e^{-\frac{c_{1}e_{a}^{}}{w_{3}\rho }}}{e_{a}^{}w_{4}c_{1}+w_{3}c_{2}}+\frac{c_{1}c_{2}\left(w_{3}-e_{a}^{}w_{4}\right)e^{-\frac{e_{a}^{}\left(c_{1}+c_{2}\right)}{\rho (w_{3}-e_{a}^{}w_{4})}}}{\left(e_{a}^{}w_{4}c_{1}+w_{3}c_{2}\right)\left(c_{1}+c_{2}\right)}. \end{array}$$

If $w_{3}-e_{a}^{}w_{4}<0$, with the similar approaches, we have

(36) $$P_{a-r}^{1}=\frac{c_{2}}{c_{1}+c_{2}}-\frac{w_{3}c_{2}}{e_{a}^{}w_{4}c_{1}+w_{3}c_{2}}e^{-\frac{e_{a}^{}c_{1}}{w_{3}\rho }}$$

Case 2: when X<Y, $x_{a}$ can be decoded only after $x_{b}$ is decoded correctly, so we should derive the OP of $x_{b}$ first, and then obtain the OP of $x_{a}$. The OP of $x_{b}$ is expressed by

(37) $$\begin{array}{cc} P_{b-r}^{2} & =\mathrm{Pr}\left\{\gamma_{b-r}^{2}<e_{b}^{}\right\}\\ & =\mathrm{Pr}\{Y<\frac{e_{b}^{}w_{3}X}{w_{4}}+\frac{e_{b}^{}}{w_{4}\rho }\}. \end{array}$$

Considering X<Y and $Y<\frac{e_{b}^{}w_{3}X}{w_{4}}+\frac{e_{b}^{}}{w_{4}\rho }$, if $w_{4}-e_{b}^{}w_{3}>0$, by some integral operations,

(38) $$P_{b-r}^{2}=\frac{c_{2}}{c_{1}+c_{2}}+\frac{\left(e_{b}^{}w_{3}-w_{4}\right)c_{1}c_{2}}{\left(e_{b}^{}w_{3}c_{2}+w_{4}c_{1}\right)\left(c_{1}+c_{2}\right)}e^{-\frac{e_{b}^{}\left(c_{1}+c_{2}\right)}{\rho (w_{4}-e_{b}^{}w_{3})}}.$$

If $w_{4}-e_{b}^{}w_{3}<0$,

(39) $$P_{b-r}^{2}=\frac{c_{2}}{c_{1}+c_{2}}.$$

The OP of B->R at X<Y can be summarized in (40),

(40) $$P_{b-r}^{2}=\left\{\begin{array}{c} \frac{c_{2}}{c_{1}+c_{2}}\;\;\;\;\;\;\;\;\;\;\;\;\;\;\;\;\;\;\;\;\;\;\;\;\;\;\;\;\;\;\;\;\;\;\;\;\;\;\;\;\;\;\;\;\;\;\;\;\;\;\;\;\;\;\;\;\;\;\;\;\;\;\;\;\;\;\;\;\;\;\;\;\;\;\;\;\;\;\;\;\;\;\;\;\;\;\;\;\;\;\;\;\;\;\;\;\;\;\;\;\;\;\;\;\;\;\;\;\;\;\;\;\;\;\;\;\;\;\;\;\;if\;\;w_{4}-e_{b}^{}w_{3}<0\;\\ \frac{c_{2}}{c_{1}+c_{2}}+\frac{\left(e_{b}^{}w_{3}-w_{4}\right)c_{1}c_{2}e^{-\frac{e_{b}^{}\left(c_{1}+c_{2}\right)}{\rho (w_{4}-e_{b}^{}w_{3})}}}{\left(e_{b}^{}w_{3}c_{2}+w_{4}c_{1}\right)\left(c_{1}+c_{2}\right)}\;\;\;\;\;\;\;if\;\;w_{4}-e_{b}^{}w_{3}>0\; \end{array}\right.$$

Based on $P_{b-r}^{2}$, the OP of $x_{a}$ is given by

(41) $$P_{a-r}^{2}=1-\left(1-\mathrm{Pr}\left\{\gamma_{a-r}^{2}<e_{a}^{}\right\}\right)\times \left(1-P_{b-r}^{2}\right)$$

Apply the result from (10), (41) is expressed as

(42) $$P_{a-r}^{2}=1-e^{-\frac{e_{a}^{}c_{1}}{w_{3}\rho }}\times \left(1-P_{b-r}^{2}\right)$$

where $P_{b-r}^{2}$ is shown in (40).

To summarize, $P_{a-r}$ is shown in (43).

(43) $$P_{a-r}^{}=\left\{\begin{array}{cc} \frac{c_{2}}{c_{1}+c_{2}}-\frac{w_{3}c_{2}}{e_{a}^{}w_{4}c_{1}+w_{3}c_{2}}e^{-\frac{c_{1}e_{a}^{}}{w_{3}\rho }}+\frac{c_{1}c_{2}\left(w_{3}-e_{a}^{}w_{4}\right)}{\left(e_{a}^{}w_{4}c_{1}+w_{3}c_{2}\right)\left(c_{1}+c_{2}\right)}e^{-\frac{e_{a}^{}\left(c_{1}+c_{2}\right)}{\rho (w_{3}-e_{a}^{}w_{4})}} & if\;\;w_{3}-e_{a}^{}w_{4}>0\;\&\;X>Y\\ \frac{c_{2}}{c_{1}+c_{2}}-\frac{w_{3}c_{2}}{e_{a}^{}w_{4}c_{1}+w_{3}c_{2}}e^{-\frac{e_{a}^{}c_{1}}{w_{3}\rho }} & if\;\;w_{3}-e_{a}^{}w_{4}<0\;\&\;X>Y\\ \left(1-e^{-\frac{e_{a}^{}c_{1}}{w_{3}\rho }}\right)\left(\frac{c_{1}}{c_{1}+c_{2}}-\frac{\left(e_{b}^{}w_{3}-w_{4}\right)c_{1}c_{2}}{\left(e_{b}^{}w_{3}c_{2}+w_{4}c_{1}\right)\left(c_{1}+c_{2}\right)}e^{-\frac{e_{b}^{}\left(c_{1}+c_{2}\right)}{\rho (w_{4}-e_{b}^{}w_{3})}}\right) & if\;\;w_{4}-e_{b}^{}w_{3}>0\;\;\&\;X<Y\\ \left(1-e^{-\frac{e_{a}^{}c_{1}}{w_{3}\rho }}\right)\times \frac{c_{1}}{c_{1}+c_{2}} & if\;\;w_{4}-e_{b}^{}w_{3}<0\;\;\&\;\;X<Y \end{array}\right.$$

#### 3.2.2. Outage Probability of Link R->D

When successful decoding the signal from A at phase 2, R forwards $x_{a}$ with the power harvested from phase 1. Upon receiving the combined signal, D decodes it using NOMA. Since D should first decode and remove the strong signal $x_{b}$ correctly, therefore, the OP of $x_{a}$ at D is expressed by

(44) $$P_{r-d}^{}=1-\left(1-\underset{\left[II\right]}{\underbrace{\mathrm{Pr}\{\gamma_{r-d}^{3}<e_{a}^{}\}}}\right)\times \left(1-P_{b-d}^{3}\right)$$

where $P_{b-d}^{3}$ is shown in (31).

Utilizing (16) and the PDF of *Z*, [II] in (44) is derived as

(45) $$\left[II\right]=1-\underset{\left[III\right]}{\underbrace{\int_{0}^{\infty }e^{-\frac{e_{a}^{}\left(1-\beta \right)c_{3}}{2\eta \beta \rho V}}f_{V}\left(v\right)dv}}.$$

If $c_{2}w_{1}-c_{1}w_{2}=0$,

(46) $$\left[III\right]=\frac{c_{1}c_{2}}{w_{1}w_{2}}\int_{0}^{\infty }ve^{-\frac{e_{a}^{}\left(1-\beta \right)c_{3}}{2\eta \beta \rho v}-\frac{c_{2}v}{w_{2}}}dv$$

Define $s=\frac{c_{2}v}{w_{2}}$ and $b=\frac{e_{a}^{}\left(1-\beta \right)c_{2}c_{3}}{2\eta \beta \rho w_{2}}$,

(47) $$\begin{array}{cc} \left[III\right] & =\frac{w_{2}c_{1}}{w_{1}c_{2}}\int_{0}^{\infty }se^{-s}e^{-b/s}ds\\ & =\frac{w_{2}c_{1}}{w_{1}c_{2}}\Gamma_{b}\left(2\right) \end{array}$$

where $\Gamma_{b}\left(2\right)$ is generalized incomplete gamma function.

According to the equation $\Gamma_{b}\left(a\right)=2b^{a/2}K_{a}\left(2\sqrt{b}\right)$ [26],

(48) $$\left[III\right]=\frac{e_{a}^{}\left(1-\beta \right)c_{1}c_{3}}{\eta \beta \rho w_{1}}K_{2}\left(\sqrt{\frac{2e_{a}^{}\left(1-\beta \right)c_{2}c_{3}}{\eta \beta \rho w_{2}}}\right)$$

where $K_{2}\left(\cdot \right)$ is the second order modified Bessel function of the second kind.

Hence, at $\;c_{2}w_{1}-c_{1}w_{2}=0$,

(49) $$P_{r-d}^{}=1-\left(1-P_{b-d}^{3}\right)\times \frac{e_{a}^{}\left(1-\beta \right)c_{1}c_{3}}{\eta \beta \rho w_{1}}K_{2}\left(\sqrt{\frac{2e_{a}^{}\left(1-\beta \right)c_{2}c_{3}}{\eta \beta \rho w_{2}}}\right)\;\;\;$$

If $c_{2}w_{1}-c_{1}w_{2}\neq 0$,

(50) $$\begin{array}{c} \left[III\right]=\frac{c_{1}c_{2}}{c_{2}w_{1}-c_{1}w_{2}}\times \left(\underset{\left[IV\right]}{\underbrace{\int_{0}^{\infty }e^{-\frac{e_{a}^{}\left(1-\beta \right)c_{3}}{2\eta \beta \rho v}-\frac{c_{1}v}{w_{1}}}dv}}-\underset{\left[V\right]}{\underbrace{\int_{0}^{\infty }e^{-\frac{e_{a}^{}\left(1-\beta \right)c_{3}}{2\eta \beta \rho v}-\frac{c_{2}v}{w_{2}}}dv}}\right) \end{array}$$

According to the equation $\int_{0}^{\infty }e^{-\frac{p}{4x}-qx}dx=\sqrt{\frac{p}{q}}K_{1}\left(\sqrt{pq}\right)$,

(51) $$\left[IV\right]=\frac{w_{1}}{c_{1}}\sqrt{\frac{2e_{a}^{}\left(1-\beta \right)c_{1}c_{3}}{\eta \beta \rho w_{1}}}K_{1}\left(\sqrt{\frac{2e_{a}^{}\left(1-\beta \right)c_{1}c_{3}}{\eta \beta \rho w_{1}}}\right)$$

and

(52) $$\left[V\right]=\frac{w_{2}}{c_{2}}\sqrt{\frac{2e_{a}^{}\left(1-\beta \right)c_{2}c_{3}}{\eta \beta \rho w_{2}}}K_{1}\left(\sqrt{\frac{2e_{a}^{}\left(1-\beta \right)c_{2}c_{3}}{\eta \beta \rho w_{2}}}\right)$$

where $K_{1}\left(\cdot \right)$ is the first order modified Bessel function of the second kind.

Hence, we have at $c_{2}w_{1}-c_{1}w_{2}\neq 0$,

(53) $$\begin{array}{c} P_{r-d}^{}=1-\frac{\left(1-P_{b-d}^{3}\right)c_{1}c_{2}}{c_{2}w_{1}-c_{1}w_{2}}\times \;\;\left[\frac{w_{1}}{c_{1}}\sqrt{\frac{2e_{a}^{}\left(1-\beta \right)c_{1}c_{3}}{\eta \beta \rho w_{1}}}K_{1}\left(\sqrt{\frac{2e_{a}^{}\left(1-\beta \right)c_{1}c_{3}}{\eta \beta \rho w_{1}}}\right)-\frac{w_{2}}{c_{2}}\sqrt{\frac{2e_{a}^{}\left(1-\beta \right)c_{2}c_{3}}{\eta \beta \rho w_{2}}}K_{1}\left(\sqrt{\frac{2e_{a}^{}\left(1-\beta \right)c_{2}c_{3}}{\eta \beta \rho w_{2}}}\right)\right]\;\;\;\;\; \end{array}$$

#### 3.2.3. Outage Probability of the Whole Link A->D

Based on the OP of link A->R and R->D, it is easy to obtain the OP of link A->D,

(54) $$P_{a-d}=1-\left(1-P_{a-r}\right)\times \left(1-P_{r-d}\right)$$

where $P_{a-r}$ is shown in (43), and $P_{r-d}$ is shown in (49) at $c_{2}w_{1}-c_{1}w_{2}=0$ and (53) at $c_{2}w_{1}-c_{1}w_{2}\neq 0$.

### 3.3. System Throughput

During a unit transmission period, the system throughput from B to D is the sum of throughput at three phases, which is given by

(55) $$S_{b-d}=\beta r_{b}\left(1-P_{b-d}^{1}\right)+\frac{1-\beta }{2}r_{b}\left(1-P_{b-d}^{2}\right)+\frac{1-\beta }{2}r_{b}\left(1-P_{b-d}^{3}\right)$$

Since the effective transmission period from A to D is (1−β)/2, the system throughput of link A->D is given by

(56) $$S_{a-d}=\frac{1-\beta }{2}r_{a}\left(1-P_{a-d}^{}\right)$$

As a result, the overall system throughput of our uplink NOMA model is expressed by

(57) $$\begin{array}{cc} S_{N} & =S_{b-d}+S_{a-d}\\ & =\beta r_{b}\left(1-P_{b-d}^{1}\right)+\frac{1-\beta }{2}r_{b}\left(1-P_{b-d}^{2}\right)+\frac{1-\beta }{2}r_{b}\left(1-P_{b-d}^{3}\right)+\frac{1-\beta }{2}r_{a}\left(1-P_{a-d}^{}\right) \end{array}$$

## 4. Numerical Results

In this section, numerical results are presented to facilitate understanding the performance of our wireless powered relay model with uplink NOMA. Monte Carlo simulations are performed to verify our analysis. In the numerical simulation, we select two values of distance from A to R (i.e., $d_{1}=1.5$ m and $d_{1}=3$ m) to investigate the system performance in two cases, i.e., X>Y and X<Y, which have been discussed theoretically in Section 3. Since there are different OPs at uplink communication phases, in the following figures we only illustrate those OP curves impacted by the parameter discussed in this subsection. We also compare the performance of our proposed TS-based EH scheme, where relay harvests energy from two nodes, with the traditional TS-based EH scheme, where relay harvests energy just from the far-end source node. Furthermore, we also exhibit the differences in system throughput between NOMA and TDMA-based OMA. In order to distinguish OP curves in each phase and system throughput curves in different communication fashions, we make descriptions for each curve in Table 1. In addition, the default values of system parameters and their corresponding descriptions related to our simulation are all listed in Table 2.

### 4.1. OP and System Throughput vs. Power Allocation Factor $w_{1}$ and $w_{3}$

In this subsection, we consider the variations on OPs and system throughput when $w_{1}$ or $w_{3}$ ranges from 0.1 to 0.9.

**Figure 2:**
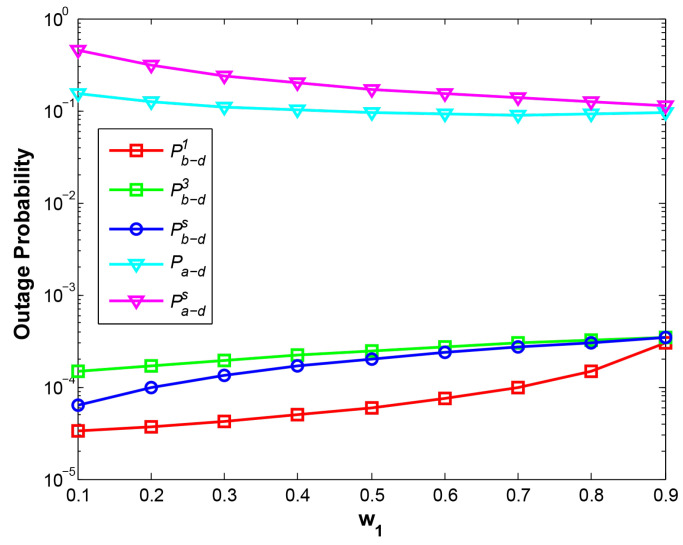
OP vs. $w_{1}$ at $d_{1}=1.5$.

**Figure 3:**
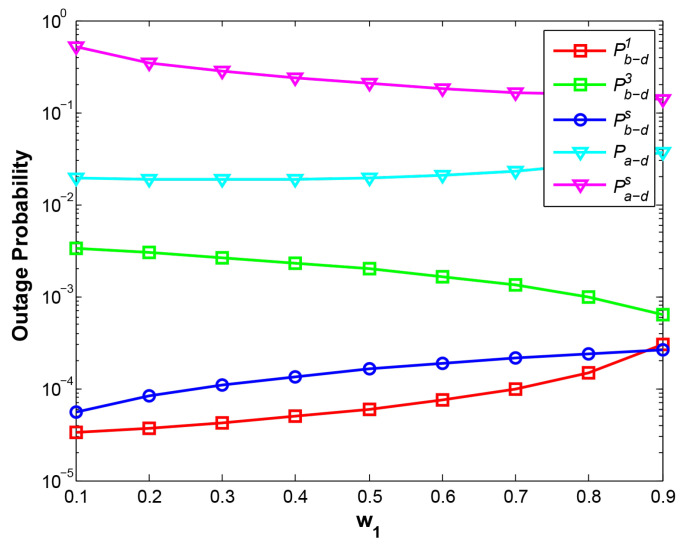
OP vs. $w_{1}$ at $d_{1}=3$.

With the larger $w_{1}$, the near-end node B is allocated less transmitting power, which leads to greater OP of B->D at phase 1 at two cases, i.e., at $d_{1}$ = 1.5 shown in Figure 2 and at $d_{1}$ = 3 shown in Figure 3. However, two curves $P_{a-d}$ and $P_{b-d}^{3}$ demonstrate the opposite results, where the curve $P_{a-d}$ drops at $d_{1}=1.5$ and rises at $d_{1}=3$, while $P_{b-d}^{3}$ rises at $d_{1}=1.5$ and drops at $d_{1}=3$. When the relay R is close to node A ($d_{1}=1.5$), R can harvest more power with increasing $w_{1}$, therefore, the higher power relay signal results in the lower $P_{a-d}$. On the contrary, when R is close to B ($d_{1}$ = 3), with rising $w_{1}$, R harvests less power from the adjacent node B, which leads to the higher $P_{a-d}$. The reason for different results of $P_{b-d}^{3}$ at two cases is that the stronger signal of A means the stronger interference with B and the higher value of $P_{b-d}^{3}$, and vice versa. As a result, $w_{1}$ should be carefully considered according to the position of relay node to achieve the required system OPs of two nodes.

**Figure 4:**
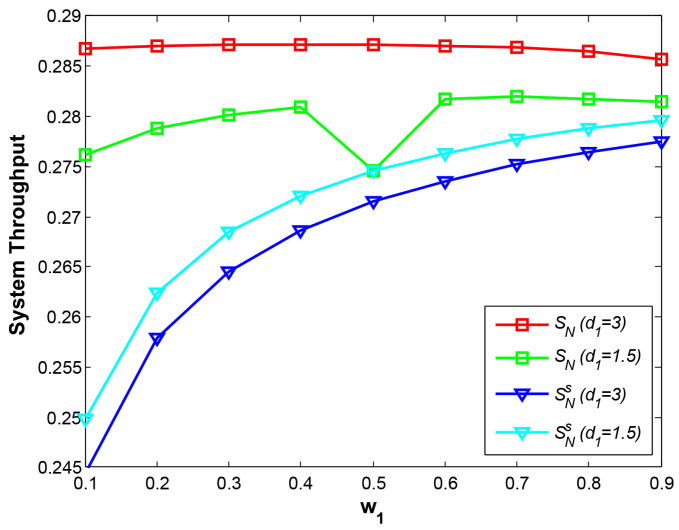
System throughput vs. $w_{1}$.

Compared with the traditional EH scheme where relay harvests the energy only from a source, our EH scheme results in a higher-power relay signal for the far-end node, hence, the OP of A->D and total system throughput have been greatly improved at the expense of slight degradation for the OP of B->D, which is also illustrated in Figure 4.

From Figure 4, it is obvious that $S_{N}$ outperforms $S_{N}^{s}$ greatly at different $w_{1}$ and different positions of the relay node, illustrating the benefits from our EH scheme. Additionally, with our EH scheme the system throughput at $d_{1}=3$ is much larger than that at $d_{1}=1.5$. When R is close to B, R still obtains plenty of energy if more energy is allocated to B at smaller $w_{1}$. The most important reason is that path fading of link R->D at $d_{1}=3$ is much smaller than at $d_{1}=1.5$, leading to lower OP and higher throughput. Therefore, the maximum value on the curve $S_{N}$ ($d_{1}=3$) occurs at $w_{1}=0.3$ representing the 70% of total power for node B. Similarly, the maximum value on the curve $S_{N}$ ($d_{1}=1.5$) occurs at $w_{1}=0.7$. We also observe that the lowest value on the curve $S_{N}$ ($d_{1}=1.5$) occurs at $w_{1}=0.5$. The reason is that R harvests the least energy from two nodes when total power is allocated equally for two nodes. Additionally, it is difficult for R to decode the signal A from the combined signal with NOMA when the SNRs of the signal A and B at R are close, which also reduces the system throughput.

Under the traditional EH scheme, it is observed that system throughput at $d_{1}=1.5$ outperforms that at $d_{1}=3$. Due to energy harvesting only from one source node, relay R closer to the source node can obtain more power even though a longer distance from R to D causes deeper path loss. In general, our proposed EH scheme can achieve the highest throughput, where the relay node close to the near-end node not only harvest more energy but also leads to less path fading from R to D.

**Figure 5:**
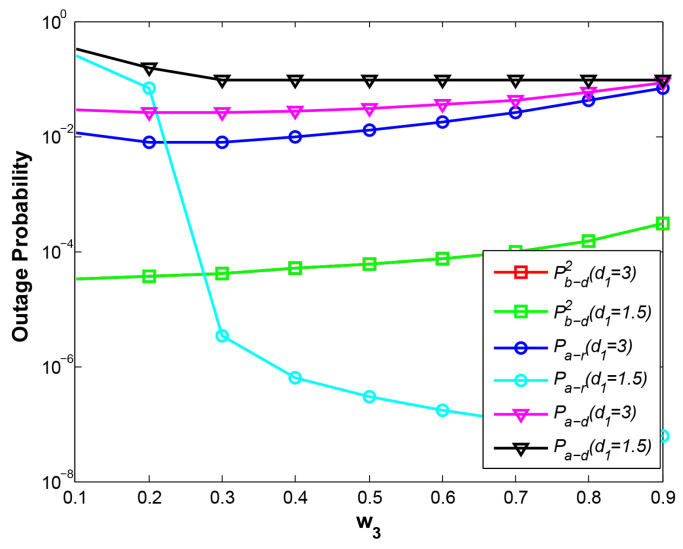
OP vs. $w_{3}$.

The parameter $w_{3}$ is the power allocation factor at phase 2, so it has no impact on the OPs of B->D at phase 1 and phase 3, which are not shown in Figure 5. With $w_{3}$ increasing, the OPs of B->D at phase 2 at two cases rise identically due to less power assigned for B and two curves $P_{b-d}^{2}$ ($d_{1}=1.5$) and $P_{b-d}^{2}$ ($d_{1}=3$) coincide with each other. In terms of OPs of links A->R and A->D at $d_{1}=1.5$, the stronger transmitting signal from A leads to higher successful decoding rate at R and gains much lower OP of A->R. Hence, the curve $P_{a-r}$ ($d_{1}=1.5$) descends rapidly with larger $w_{3}$. However, the curves $P_{a-d}$ ($d_{1}=1.5$) declines slowly, especially at bigger $w_{3}$. The reason is that the OP of link R->D, which depends on the energy harvested at phase 1, is much larger than the OP of A->R, indicating that the OP of link A->D is determined by the OP of link R->D mainly. Additionally, at $d_{1}=3$, it is observed that the OP of A->R declines and obtains a minimum value at $w_{3}$ = 0.2 but rise with larger $w_{3}$. When R is closer to B, R decodes the strong signal from B first, and then decodes the weak signal A. Hence, if $w_{3}$ is bigger, the stronger transmitting signal from A will prevent the decoding the signal B at R, and finally result in higher OP of A->R. Moreover, if $w_{3}$ is smaller, weak signal of A also causes higher OP of A->R. Similarly, the OP of A->D presents the same result as the OP of A->R.

**Figure 6:**
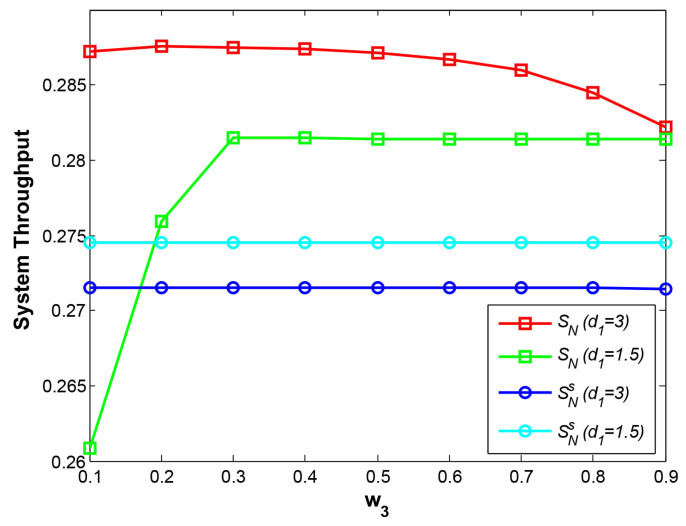
System throughput vs. $w_{3}$.

In Figure 6, the system throughput curves at different cases are shown. The performance with the traditional EH scheme is clearly poor and the curves of $S_{N}^{s}$ at two relay positions are basically stable and far below the curves of $S_{N}$ due to lower energy harvested. Furthermore, The reason of performance degradation at the left side of the curves $S_{N}$ ($d_{1}=1.5$) and the right side of the curves $S_{N}$ ($d_{1}=3$) lies in higher OPs shown in Figure 5 discussed above. In general, it is obvious that $S_{N}$ outperforms $S_{N}^{s}$ at different relay positions if a proper $w_{3}$ value is given. Moreover, the best system throughput with our EH scheme at $d_{1}=3$ is achieved mainly due to the lower path fading between R and D.

### 4.2. OP and System Throughput vs. Time Switching Factor β

**Figure 7:**
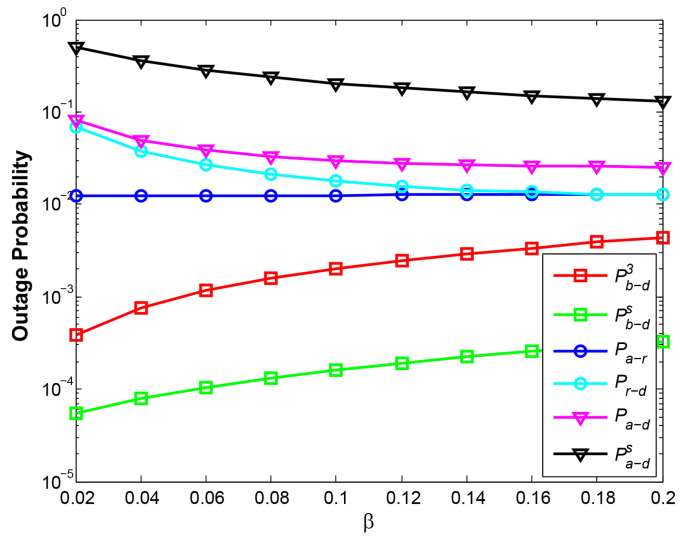
OP vs. β at $d_{1}=3$.

Figure 7 shows the relationship between OP and β at $d_{1}=3$. We do not show the case at $d_{1}=1.5$ because the two cases both reflect the approximate results. With larger β, the relay signal with more harvested energy from R will interfere with the transmission of B->D at phase 3. That is why the curve $P_{b-d}^{3}$ increases gradually. Additionally, the curve $P_{a-r}$ is also observed to rise slightly. The reason is that larger β will reduce effective transmission time. Thus, at the same RTR and transmitting power for A by $w_{3}$, shorter transmission time results in the higher OP of A->R. Moreover, although shorter effective transmission time may also increase the OP of R->D, more energy harvested at R decreases the OP of R->D. Therefore, the curves $P_{r-d}$ and $P_{a-d}$ decline gradually at the given reasonable range of β from 0.02 to 0.2. Additionally, the reason that $P_{a-d}$ and $P_{b-d}^{s}$ outperform $P_{a-d}^{s}$ and $P_{b-d}^{3}$, respectively, lies in different EH schemes.

**Figure 8:**
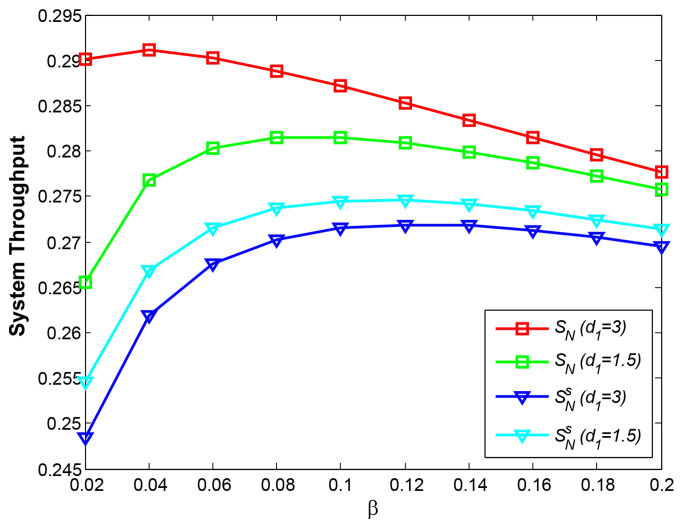
System throughput vs. β at $d_{1}=3$.

Figure 8 shows the relationship between system throughput and β. The curve $S_{N}$ ($d_{1}=3$) illustrates the best system performance among all curves. When β is smaller, the energy harvested by R is lower, and when β is greater, the effective transmission duration becomes shorter. These two cases both lead to the lower system throughput. Therefore, a maximum value in each curve exists at a certain β. The curves $S_{N}$ ($d_{1}=3$) and $S_{N}$ ($d_{1}=1.5$) gain their maximum values at β = 0.04 and β = 0.08, respectively. In addition, the reason for a larger β at $d_{1}=1.5$ than $d_{1}=3$ is due to more energy required against long distance fading.

### 4.3. OP and System Throughput vs. Distance $d_{1}$ between A and R

**Figure 9:**
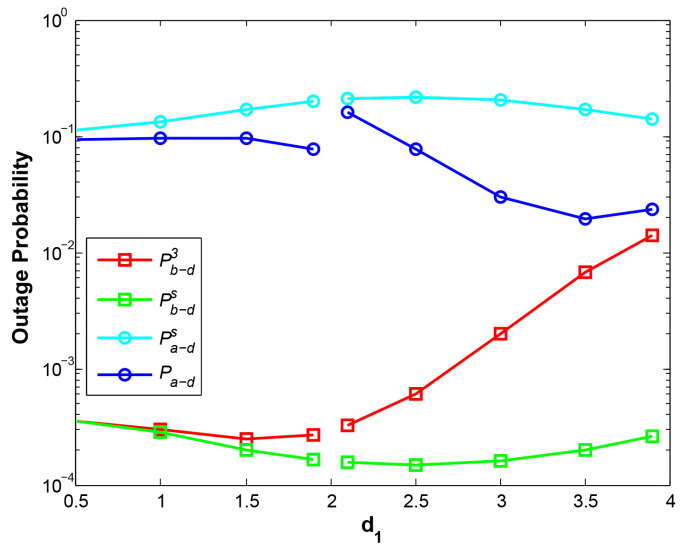
OP vs. $d_{1}$.

Figure 9 plots the OP performance with different positions of relay. It is noted that we select $d_{1}=3.9$ m instead of $d_{1}=4$ m because it is impossible for R to stay with B. Furthermore, NOMA cannot successfully decode two combined signals when they are similar path loss gain, i.e., $d_{1}=2$, so we remove the points $d_{1}=2$ at the curves. Since we have discussed two cases $d_{1}$ < $d_{2}$ and $d_{1}$ > $d_{2}$ in Section 3.2, accordingly, we select two values $d_{1}=1.9$ representing case 1 ($d_{1}$ < $d_{2}$) and $d_{1}=2.1$ representing case 2 ($d_{1}$ > $d_{2}$) for investigation in this simulation.

It is observed that the curves of $P_{b-d}^{3}$ and $P_{a-d}$ are limited within the range of curves of $P_{b-d}^{s}$ and $P_{a-d}^{s}$, which explains that with our EH scheme the OP of the far-end node is improved at the cost of trivial performance degradation for the near-end node. When relay R is closer to A, higher OP of A->D is gained due to deeper path fading from R to D, while the OP of B->D is very lower due to minor interference from R. When R approaches B, the link A->D gains lower OP due to smaller fading of link R->D, as well as higher power harvested from B correspondingly. The curve OP of B->D becomes larger due to stronger interference from R. It is noted that when R is in the middle of two nodes A and B, i.e., at $d_{1}=2.1$, due to approximate signal strength from A and B, NOMA decoding at R will cause higher OP. Furthermore, when R is getting closer to B, e.g., $d_{1}>3.5$, the OP of A->D becomes bigger again. That is because the higher OP of B->D prevents the decoding of signal A.

**Figure 10:**
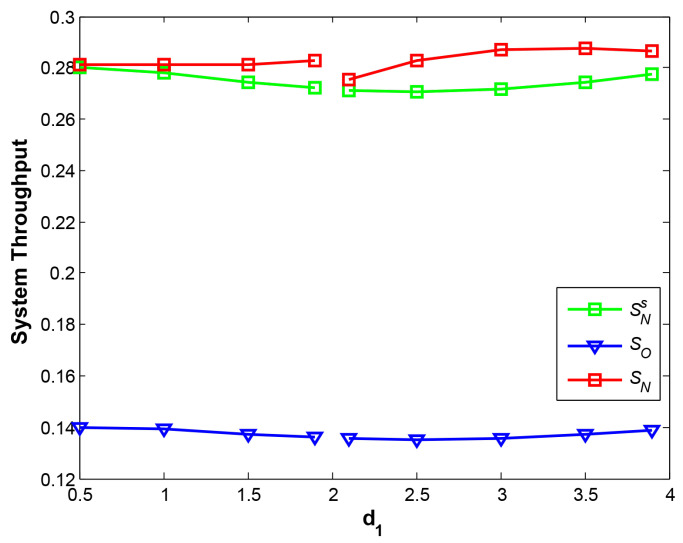
System throughput vs. $d_{1}$.

Figure 10 shows the curves of system throughput at three cases. The first is NOMA with our proposed EH scheme, the second is NOMA with the traditional EH scheme, and the last is TDMA-based OMA with the traditional EH scheme. Obviously, NOMA at two cases achieves about twice system throughput compared with regular OMA, demonstrating a huge advantage of NOMA over OMA. In addition, our EH scheme outperforms the traditional EH scheme, and the maximum value is obtained at $S_{N}$ at $d_{1}=3.5$, which indicates the position of relay plays a significant role on overall system throughput.

### 4.4. OP and System Throughput vs. Total Transmitting Power p

**Figure 11:**
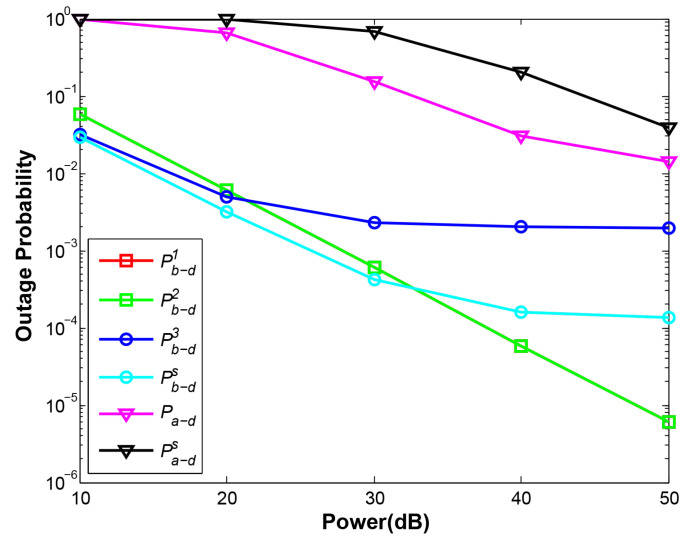
OP vs. total transmitting power.

**Figure 12:**
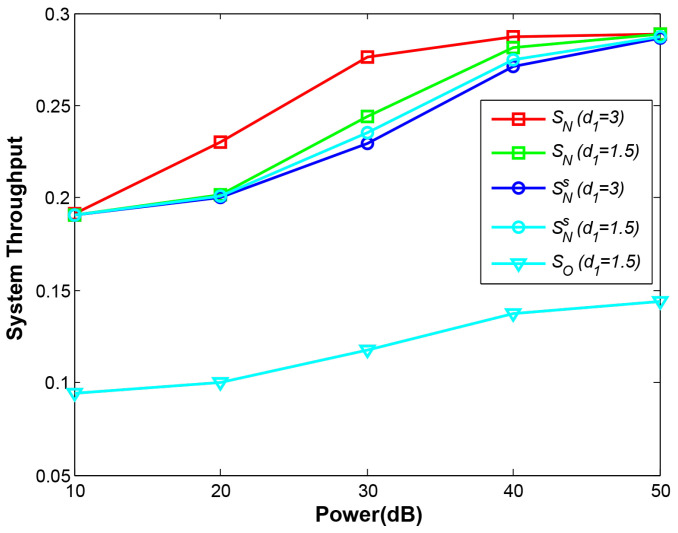
System throughput vs. total transmitting power.

Figure 11 plots OPs under different transmitting power from 10 dB to 50 dB. All the OP curves definitely decrease given the larger transmitting power. At phase 1 and phase 2, the near-end node B can communicate with D regularly without being affected by the far-end node A, therefore, the curves $P_{b-d}^{1}$ and $P_{b-d}^{2}$ both decline rapidly (two curves are overlapped in the figure). However, at larger power given, the curves $P_{b-d}^{3}$ and $P_{b-d}^{s}$ tend to decline slowly. That is because greater power not only increases the SNR of B but also increases the SNR of A, which is interference with D to decode combined signal of A and B. One can infer that more power supply may not improve the OP of near-end node efficiently in NOMA. The conclusion can also be drawn from Figure 12. In this figure, the best performance of NOMA can be observed at $d_{1}=3$ under our proposed EH scheme, but with the larger power supply, all the curves except OMA converge gradually, showing that under different EH schemes and different relay positions NOMA achieves an approximate system throughput. The results also indicate NOMA plays the crucial role with our proposed EH scheme at the constrained power supply. Moreover, NOMA still demonstrates excellent system performance against OMA. One can infer that in energy-constrained IoT uplink networks, NOMA is a better selection than OMA.

## 5. Conclusions

In this paper, we investigate wireless powered relay with uplink NOMA in IoT networks to improve system performance, as well as reduce energy consumption. The closed-form expressions of OPs of uplink communication and overall system throughput are derived. Our numerical simulation demonstrates superior system performance by optimizing important parameters, e.g., TS factor, power allocation factor, the position of relay node at the constrained total transmitting power compared to the traditional EH scheme and OMA. The most important conclusion is that with our EH scheme, by optimizing relay position more energy is harvested from the near-end node at the relay and long distance relay signal attenuation from the relay to the destination is greatly reduced as well, which achieves the best system performance. Additionally, although TS relay communication reduces the effective transmission time compared with PS relay communication, adequate energy harvested from two transmitting nodes in our NOMA model can offset the effect and gain better performance.

## Figures and Tables

**Table 1:** Curves Description.

Curves Name	Description
$P_{b-d}^{1}$	OP of B->D at phase 1
$P_{b-d}^{2}$	OP of B->D at phase 2
$P_{b-d}^{3}$	OP of B->D at phase 3 with our proposed EH scheme
$P_{b-d}^{s}$	OP of B->D at phase 3 with the traditional EH scheme
$P_{a-r}$	OP of A->R with our proposed EH scheme
$P_{r-d}$	OP of R->D with our proposed EH scheme
$P_{a-d}$	OP of A->D with our proposed EH scheme
$P_{a-d}^{s}$	OP of A->D with the traditional EH scheme
$S_{N}$	NOMA System throughput with our proposed EH scheme
$S_{N}^{s}$	NOMA System throughput with the traditional EH scheme
$S_{O}^{s}$	OMA System throughput with the traditional EH scheme

**Table 2:** Simulation Parameters by Default.

Parameters	Description
η=1	energy conversion coefficient
α=3	path loss exponent
d=5 m	distance between A and D
$d_{1}=1.5$ or 3 m	distance between A and R
$d_{4}=1$ m	distance between B and D
$d_{2}=d-d_{1}-d_{4}$	distance between R and B
$d_{3}=d-d_{1}$	distance between R and D
β=0.1 s	time switching factor
$r_{a}=0.2$ bit/s/Hz	required target rate of A->D
$r_{b}=0.2$ bit/s/Hz	required target rate of B->D
p=40 dB	total transmitting power
$w_{1}=0.5$	power allocation factor for A at phase 1
$w_{2}=1-w_{1}$	power allocation factor for B at phase 1
$w_{3}=0.5$	power allocation factor for A at phase 2
$w_{4}=1-w_{3}$	power allocation factor for B at phase 2

## Appendix A

**Proof of Theorem** **1.** Here we derive the PDF of $V=V_{1}+V_{2}$ where $V_{1}=w_{1}X$ and $V_{2}=w_{2}Y$. Due to $X∼E\;(\lambda_{1}=\frac{1}{c_{1}})$ and $Y∼E\;(\lambda_{2}=\frac{1}{c_{2}})$, we have $V_{1}=w_{1}X∼E\;\left(\frac{w_{1}}{c_{1}}\right)$ and $V_{2}=w_{2}X∼E\;\left(\frac{w_{2}}{c_{2}}\right)$. Since $V_{1}$ and $V_{2}$ are independent, the PDF of V is calculated by

$$\begin{array}{cc} f_{V}(v) & =\int_{-\infty }^{\infty }f_{V_{1}}\left(v_{1}\right)f_{V_{2}}(v-v_{1})dv_{1}\\ & =\frac{c_{1}c_{2}}{w_{1}w_{2}}e^{-\frac{c_{2}v}{w_{2}}}\int_{0}^{v}e^{\frac{c_{2}w_{1}-c_{1}w_{2}}{w_{1}w_{2}}v_{1}}dv_{1} \end{array}$$

If $c_{2}w_{1}-c_{1}w_{2}=0$, we have

$$f_{V}\left(v\right)=\frac{c_{1}c_{2}v}{w_{1}w_{2}}e^{-\frac{c_{2}v}{w_{2}}}$$

else

$$\begin{array}{cc} f_{V}(v) & =\frac{c_{1}c_{2}}{c_{2}w_{1}-c_{1}w_{2}}e^{-\frac{c_{2}v}{w_{2}}}\left(e^{\frac{c_{2}w_{1}v-c_{1}w_{2}v}{w_{1}w_{2}}}-1\right)\\ & =\frac{c_{1}c_{2}}{c_{2}w_{1}-c_{1}w_{2}}\left(e^{\frac{-c_{1}v}{w_{1}}}-e^{\frac{-c_{2}v}{w_{2}}}\right) \end{array}$$

This ends the proof. □
